# Temperature-Mediated Variations in Behavior and Mortality Caused by Non-Repellent Insecticides in Subterranean Termites (Blattodea: Rhinotermitidae)

**DOI:** 10.3390/insects10020037

**Published:** 2019-01-30

**Authors:** Franklin Y. Quarcoo, Xing Ping Hu, Arthur G. Appel

**Affiliations:** 1 Department of Entomology and Plant Pathology, Auburn University, Auburn, AL 36849-5413, USA; fquarcoo1@tuskegee.edu (F.Y.Q.); huxingp@auburn.edu (X.P.H.); 2 Department of Agricultural & Environmental Sciences, 103 Morrison-Mayberry Hall, Tuskegee University, Tuskegee, AL 36088, USA

**Keywords:** eastern subterranean termite, Formosan subterranean termite, temperature-toxicity, sublethal behaviors

## Abstract

Behavioral symptoms and mortality associated with intoxication with insecticides fipronil and indoxacarb were determined in field-collected eastern subterranean termites, *Reticulitermes flavipes* (Kollar), and Formosan subterranean termites, *Coptotermes formosanus* Shiraki. Behaviors and mortality were evaluated at three temperatures (16, 22, and 28 °C) and three concentrations of fipronil (0.5, 1, and 5 ppm) and indoxacarb (50, 75, and 100 ppm). LT_50_ (median lethal time to kill 50% of the termites) values declined with increasing concentrations and temperatures for both fipronil-exposed eastern and Formosan subterranean termites, whereas these values were not always the highest at 16 °C for indoxacarb-treated termites. The greatest change (reduction) in LT_50_ values occurred for both species between 16 and 22 °C at the lowest concentration of each insecticide. Intoxication and moribundity were the most frequently observed behaviors for fipronil-exposed termites, whereas intoxication, ataxia, and moribundity were observed for most concentration and temperature combinations for indoxacarb-exposed termites. The inherent toxicity of fipronil was higher than that of indoxacarb. The higher presence and duration of intoxication behaviors may positively affect the performance of indoxacarb against subterranean termite colonies.

## 1. Introduction

Rhinotermids construct, dwell, and forage for food in extensive and labyrinthine underground galleries, which renders a complete and uniform application of insecticides impractical [1]. The popular termite products currently used in the United States utilize non-repellent insecticides. Commonly used products are liquid, powder, granule, or foam formulations, as well as baits. Their non-repellent nature permits unknowing exposure and their slow-acting characteristics allow ample time and opportunity for termites to transfer these compounds between contaminated and naïve colony-mates through social interactions such as grooming, trophollaxis, and caregiving [2,3]. The cascading nature of this social route of distribution increases the coverage of non-repellent compounds to areas far removed from the point of application [4,5]. There are two currently EPA (USA Environmental Protection Agency) registered products containing the non-repellent active ingredients fipronil and indoxacarb, but the effect of temperature on soil termiticide performance has not been investigated.

As poikilothermic organisms, the general performance, fitness, and survival of termites is affected by environmental temperatures that alter their exposure and susceptibility to insecticides. Important life processes in termites, such as feeding, are significantly affected by temperature [6,7] and relative humidity [6]. Some researchers are, however, of the opinion that the importance of relative humidity and moisture content of food sources is greatly diminished because of the ability of subterranean termites to periodically move underground to rehydrate [8]. However, temperature is the most important abiotic factor that affects the ability of poikilothermic organisms to survive and utilize their habitat [9]. Sponsler and Appel [10] reported the critical thermal maxima and minima, as well as the upper and lower lethal limits of eastern, *Reticulitermes flavipes* (Kollar), and Formosan, *Coptotermes formosanus* Shiraki, subterranean termites. Hu and Appel [11] further reported seasonal variations of the critical thermal limits and temperature tolerance of these two termite species. Such critical limits form the basis of termite control strategies using modified temperatures, which according to Woodrow and Grace [12], have great potential. Temperature-dependent tunneling rates were reported by Smith and Rust [13]. There were similar tunneling rates in *R. hesperus* Banks exposed to temperatures between 21 and 32 °C, but a significant reduction when unacclimated termites were exposed to 15 °C. The authors proposed that this was an adaptation for foraging for food at elevated surface temperatures. Smith and Rust [14] also suggested that test insects should be held at cool temperatures and high humidity to reduce possible effects caused by the interaction of relative humidity and temperature.

Tunneling deeper into the soil to avoid cold winter surface temperatures [7,15,16,17] probably affects the performance of liquid termiticides. This is because termiticides are applied at relatively shallow depths compared with the termites’ environment, which, according to Strack and Myles [7] and Cabrera and Kamble [16], may exceed 100 cm from the soil surface. Even though the soil provides a buffer protecting subterranean termites from the extreme heat [18] and cold [19] present on the soil surface, temperature influences the general function, activity, and lifespan of a termite colony [20]. Effects of temperature on aspects of termite biology and ecology include survival [21], swarming of alates [22], oviposition, viability of eggs, and duration of incubation [23], as well as survival of gut protozoa that aid in the digestion of cellulosic food [24] and differentiation of soldiers [25].

Temperature clearly affects the toxicity of insecticides, but the direction of the effect (whether positive or negative) depends on the insect species, the insecticide, and the range of temperatures tested [26]. The rate of biochemical reactions associated with intoxication by certain insecticides is temperature-dependent. A typical example is the conversion of indoxacarb to its active metabolic form, which exerts insecticidal properties in a reaction catalyzed by esterases or amidases [27]. The efficacy of active ingredients with less toxic metabolites is compromised when the temperature accelerates their rate of metabolism [28]. The uptake and transfer of fipronil [29], noviflumuron [30], and most other active ingredients increase with temperature, with increased uptake resulting in reduced survivorship [31]. Clearance and excretion of noviflumuron is also affected significantly by temperature [30]. Earlier studies reported that temperature alone affected termite mortality even more dramatically [17,31] than the ratio of donor to recipient termites of toxicant transfer [31].

Variations in temperature between seasons, at different soil depths, and at different times of the day make research on temperature-driven variations in toxicity critical; however, studies must be conducted for specific insecticides against specific economic pests under a given set of environmental conditions. Even though fipronil is one of the most popular non-repellent compounds used in termite control [2], and indoxacarb is classified as a reduced-risk pesticide [32], there is scarce information on the specific effects of temperature on their toxicity against eastern and Formosan subterranean termites. The goal of this study was to determine the behavioral expression and mortality of eastern and Formosan subterranean termite workers exposed to fipronil and indoxacarb at different concentrations and temperatures. We hypothesized that temperature would have significant positive effects on the toxicity of both insecticides against subterranean termites.

## 2. Materials and Methods

### 2.1. Study Organism

Worker termites at the fifth larval stage at least [33] were obtained from field colonies in Auburn-Opelika, AL, and used in this study within 2 h of collection. Termites were obtained using open-bottom underground traps described by Hu and Appel [11]; traps consisted of bottomless plastic buckets 18 cm high with an internal diameter of 13 cm provisioned with corrugated cardboard rolls (15 cm high and 11 cm in diameter). Worker termites were used in these experiments because they are the most damaging caste in a colony and distribute the termiticide (through grooming, trophollaxis, and other social behaviors) to other colony members.

### 2.2. Chemicals

Insecticides used were indoxacarb (15% a.i., DuPont, Wilmington, DE) and fipronil (9.1% a.i., BASF Corp., Research Triangle Park, NC). Calculated amounts of an indoxacarb solution (ca. 1.0 mL) were applied uniformly to Whatman No. 1 filter paper (Whatman International Ltd., Maidstone, UK) placed in glass Petri dishes to obtain 50, 75, and 100 ppm indoxacarb-treated filter paper. The procedure was repeated for fipronil to obtain 0.5, 1, and 5 ppm fipronil-treated filter paper. The same stock insecticide solutions were used to treat the filter paper used against both termite species at all temperatures. Filter paper treated with distilled water was used as control treatment. Treated papers were air-dried in a laboratory hood for 24 h at about 23 °C and moistened with 0.4 mL of distilled water immediately before the introduction of termites.

### 2.3. Bioassay

Groups of 20 freshly collected eastern and Formosan subterranean termite workers, respectively, were exposed to each insecticide concentration in glass Petri dishes (5.2 cm internal diameter, 1.5 cm in height) provided with insecticide-treated Whatman No.1 filter paper of the same diameter. The Petri dishes were subsequently sealed with Parafilm^®^ (Bemis NA, Neenah, WI, USA) strips to maintain the relative humidity and prevent moisture loss, placed into transparent plastic boxes (length 29.5 cm, width 21.5 cm, height 10 cm), and maintained in incubators (Percival Scientific^®^, Perry, IA, USA) at 16, 22, and 28 °C. These temperatures were selected based on soil temperature data reported by Hu and Appel [11]. Each treatment was replicated four times with termites from four different colonies, termites were observed at 4-h intervals for the first 48 h and then at 8-h intervals until 100% mortality was recorded. All concentrations of both insecticides with both species were run simultaneously at a given temperature (in the same incubator). Thus, the results between species were directly comparable at the same temperature.

### 2.4. Observation of Behavioral Symptoms

At each 4-h interval, behavioral symptoms associated with insecticide exposure of all 20 individual termites within each replicate were video recorded. The percentage of individuals exhibiting each behavior/condition was calculated as the number of exhibiting individuals divided by the number of total termites (20) multiplied by 100. The mean percentage was calculated as the average of the four replicates. Discrete behaviors included intoxication, ataxia, and moribundity as defined by Quarcoo et al. [34], as well as death. An individual termite could only exhibit one of these behavioral symptoms or no symptom. Briefly, intoxication was defined as disorientation, horizontal oscillatory movements, and frequent changes in walking speed and direction. Ataxia included circling, walking in reverse, frequent falling, drooping antennae, and often release of stomodeal or proctodeal fluids. Termites defined as moribund were unable to move a distance equivalent to the length of their body, remained stationary on their tarsi or dorsum, and the antennae were bent and motionless.

### 2.5. Statistical Analysis

Probit analysis [35] was used to estimate the median time required to kill 50% (LT_50_) of each termite species exposed to each combination of temperature and insecticide concentration separately. Control (water only) mortality was <5% for both species at all temperatures; therefore, control mortality corrections were unnecessary [35]. The overlap of the 95% fiducial limits was used to determine significant differences in LT_50_ values between species as well as temperatures and concentrations. For each species, linear regression [35] was used to relate LT_50_ values of each concentration to exposure temperature.

## 3. Results

### 3.1. Mortality

#### 3.1.1. Fipronil

LT_50_ values ranged from 0.96 to 98.8 h for eastern subterranean termites exposed to 5.0 ppm at 28 °C and Formosan subterranean termites exposed to 0.5 ppm at 16 °C (Table 1 and Table 2). LT_50_ values declined with increasing concentrations and temperatures for both eastern (Table 1) and Formosan (Table 2) subterranean termites. The greatest change in LT_50_ values for both species occurred between 16 and 22 °C for 0.5 ppm, with values declining 68.4% and 70.5% for eastern and Formosan subterranean termites, respectively. However, regressions of LT_50_ values for each concentration over the three temperatures were not significant (*p* > 0.05), indicating that there was no linear change in LT_50_ with temperature. Based on non-overlap of the 95% fiducial limits (FL), LT_50_ values were significantly different across temperatures and concentrations between both species. The 0.5- and 1-ppm fipronil treatments had lower LT_50_ values for eastern than Formosan subterranean termites at 16 and 22 °C. At 5 ppm of fipronil, there was no difference in LT_50_ values between the termite species at any of the temperatures tested (Table 1 and Table 2).

#### 3.1.2. Indoxacarb

In contrast with the results of fipronil, treatment with indoxacarb resulted in more consistent relative susceptibility of the two species of subterranean termites; LT_50_ values indicate that indoxacarb acted more quickly against eastern than Formosan subterranean termites. LT_50_ values ranged from 7.6 h for eastern subterranean termites exposed to 100 ppm at 28 °C to 69.1 h for Formosan subterranean termites exposed to 50.0 ppm at 16 °C (Table 3 and Table 4). Moreover, unlike fipronil-treated termites, LT_50_ values for indoxacarb-treated subterranean termites were not always highest at 16 °C (Table 3 and Table 4). The highest reduction between LT_50_ values occurred for both species between 16 and 22 °C for 50 ppm; values declined 42.7% for eastern and 60.2% for Formosan subterranean termites. Similar to the results with fipronil, regressions of LT_50_ values for each concentration of indoxacarb over the three temperatures were not significant (*p* > 0.05), indicating that there was no linear change in LT_50_ with temperature.

### 3.2. Behavior

**Figure 1:**
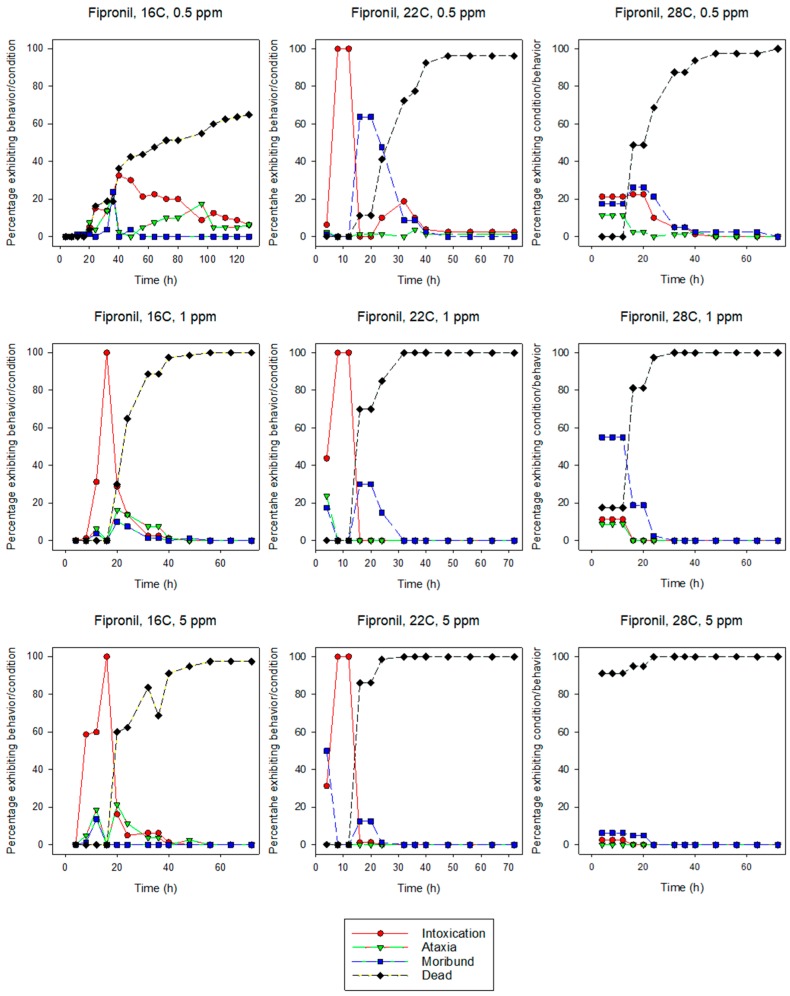
Effects of concentration and temperature on the behaviors of fipronil-exposed eastern subterranean termites, *R. flavipes*.

**Figure 2:**
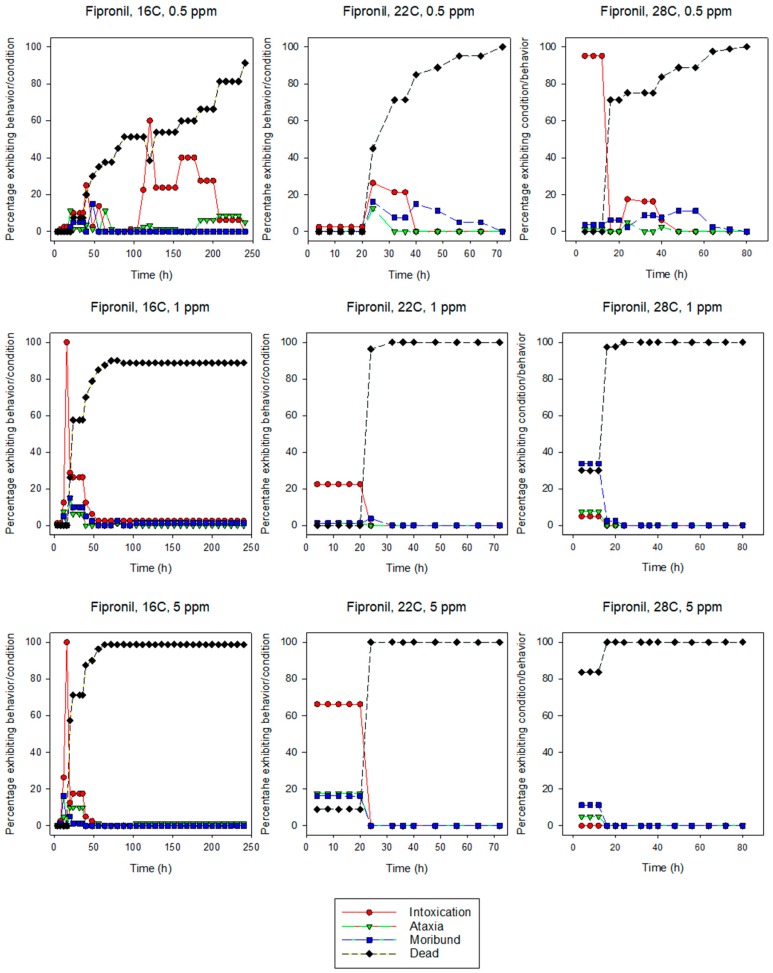
Effects of concentration and temperature on the behaviors of fipronil-exposed Formosan subterranean termites, *C. formosanus*.

**Figure 3:**
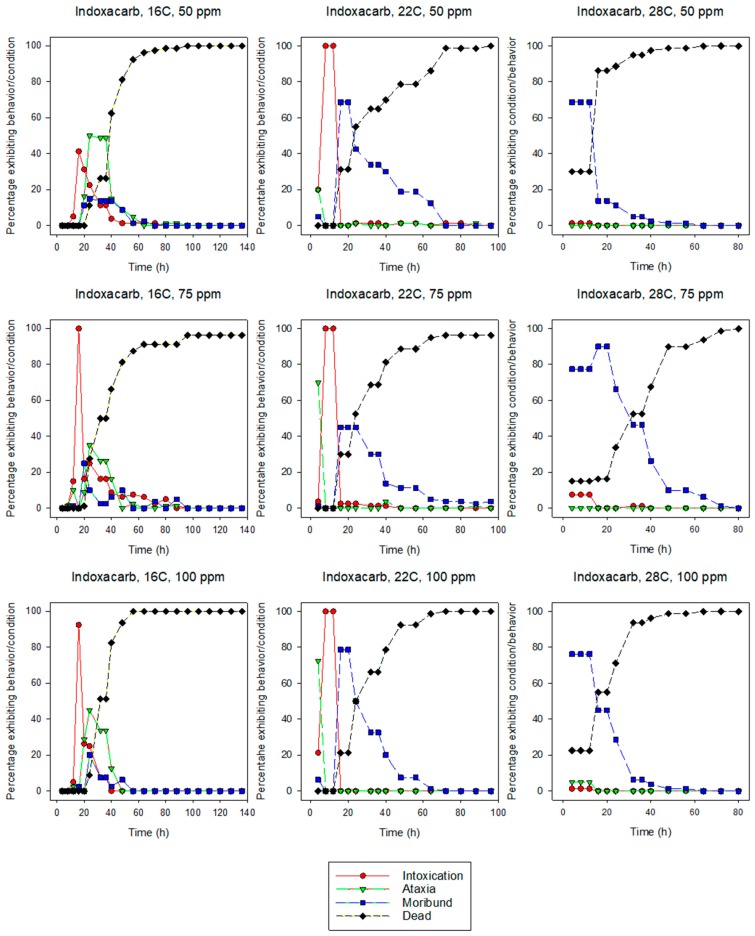
Effects of concentration and temperature on the behaviors of indoxacarb-exposed eastern subterranean termites, *R. flavipes*.

**Figure 4:**
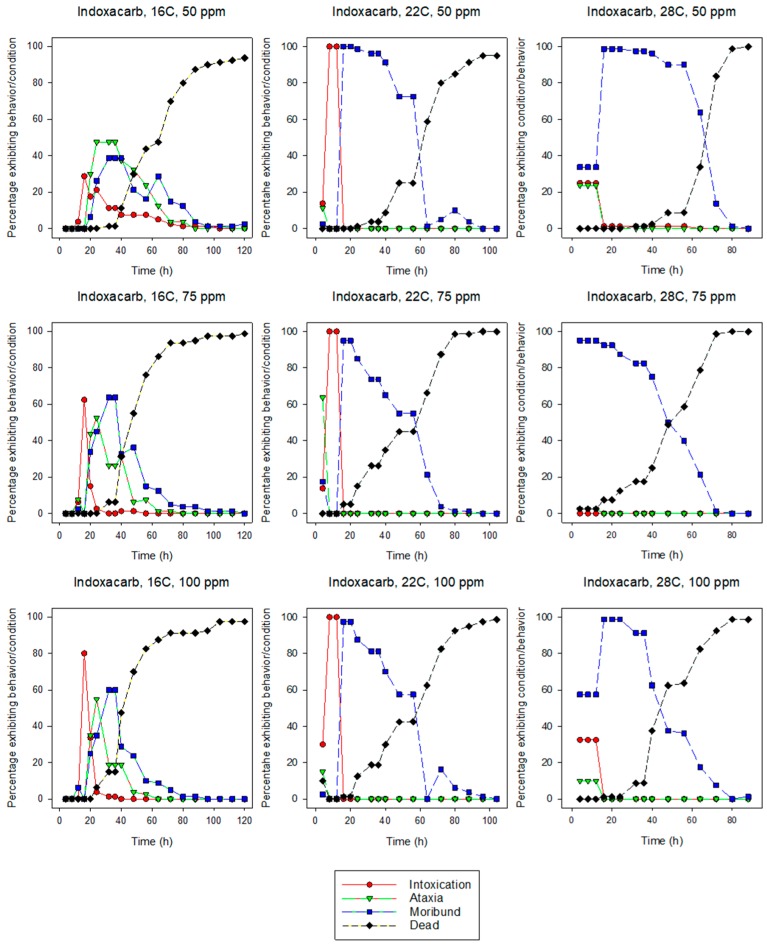
Effects of concentration and temperature on the behaviors of indoxacarb-exposed Formosan subterranean termites, *C. formosanus*.

All of the behavioral symptoms associated with insecticide exposure in termites [34] were observed for both species at most insecticide concentration and temperature combinations (Figure 1, Figure 2, Figure 3 and Figure 4). In more than half of the combinations, the sequence of behaviors was intoxication followed by ataxia, and finally moribundity prior to death. The lower the LT_50_ value (i.e., the shorter the time it took for the pesticide to cause mortality), the lower the number/variety of abnormal symptomatic behaviors observed.

Intoxication and moribundity were the most frequently observed behaviors for fipronil-exposed termites. Ataxia never reached more than about 20% for any combination of concentration and temperature. For eastern subterranean termites, intoxication ranged from 0–100%, but was generally greatest at 20 h or less and at 16 and 22 °C for both 1 and 5 ppm (Figure 1). At 28 °C, intoxication was about 20% at 0.5 ppm and declined with increasing concentrations. Moribundity ranged from 0–64% and greatest for 0.5 ppm at 22 °C, 1 ppm at 28 °C, and 5 ppm at 22 °C. The highest percentage of moribundity occurred earlier at higher concentrations. For Formosan subterranean termites, intoxication ranged from 0–100% (Figure 2). At 0.5 ppm, the maximum percentage intoxication occurred earlier at higher temperatures, while at 1 and 5 ppm it occurred earlier at lower temperatures. Moribundity was <20% for all concentration and temperature combinations except for 1 ppm and 28 °C, where moribundity was 34% at 4, 8, and 12 h (Figure 2). The fast uniform killing activity of fipronil obscured sublethal behaviors.

Intoxication, ataxia, and moribundity behaviors were observed for most concentration and temperature combinations for indoxacarb-exposed termites. For eastern subterranean termites at 16 and 22 °C, the maximum percentage of intoxication or ataxia occurred earlier than the maximal percentage of moribundity, whereas at 28 °C moribundity was the earliest and largest behavioral symptom. For Formosan subterranean termites at 16 and 22 °C, the maximum percentage of intoxication generally preceded the maximum percentage of ataxia and then moribundity. At 28 °C, the percentage of termites exhibiting moribundity exceeded all other behavioral symptoms, often reaching 100% of individuals.

## 4. Discussion

The effects of temperature on the speed of action of insecticides is particularly important for species that regularly encounter a wide range of environmental conditions. Subterranean termites in particular may be exposed to cool temperatures deep within the soil or near the soil surface during the winter, and very warm temperatures near the soil surface and within the wood they inhabit and consume during the summer.

Temperature alone can cause movement, decreased survival, rapid knock down, and mortality. In response to immediate exposure or slow cooling to 0 °C, Hu and Song [17] found that both eastern and Formosan subterranean termites rapidly moved to avoid cold temperatures; the few termites that remained at 0 °C exhibited chill coma effects, but most recovered after 5 min at ambient temperature (24 °C). In small-colony laboratory assays at constant temperature and 100% RH, Smythe and Williams [36] showed a decrease in survival of the eastern subterranean termite (from 90 to 44%) as temperature increased from 15 to 35 °C. There was 100% mortality of the eastern subterranean termite after 1 wk at 35 °C. Survival of the Formosan subterranean termite was 94–88% between 15 and 30 °C; however, there was 100% mortality after 8 wk at 35 °C. In rapidly changing temperature experiments (increases or decreases of 1 °C/min), Sponsler and Appel [10] found that the critical thermal maximum, or knock-down temperature, of worker Formosan subterranean termites (46.3 °C) was significantly greater than that of worker eastern subterranean termite (45.4 °C). The critical thermal minimum of eastern subterranean termites (13.3 °C) was significantly lower than that of Formosan subterranean termites (14.0 °C) [10]. The upper lethal limit of worker Formosan subterranean termites (48.0 °C) was significantly higher than that of worker eastern subterranean termites (46.4 °C).

In addition to having immediate physiological effects, temperature strongly influences termite behavior. Movement, tunneling behavior, foraging, and feeding of subterranean termites are affected by temperature [17,20,22,37]. Increased termite movement, particularly tunneling and food transportation, is correlated with potential damage [36]; both behaviors increase potential exposure to non-repellent soil insecticides.

Temperatures for the bioassays in this study were selected based on mean soil temperatures (at a depth of 15 cm) in Auburn Alabama [11]. The lowest soil temperature was 16 °C (February), 22 °C was the mean soil temperature in April–May and September–October, the two periods with peak termite activity; and 28 °C is within the optimal temperature range (24–35 °C) for termite feeding. Even though feeding activity is high at 35 °C, extended continuous exposure to this temperature is lethal to both eastern and Formosan subterranean termites [36]. These observations indicate that termites may briefly forage into areas of potentially lethal temperatures. The fipronil and indoxacarb concentrations used in this study were based on the efficacy results reported by Hu [38] and the abnormal behaviors, reduced movement, and tunneling behavior reported by Quarcoo et al. [5,39].

The speed of action (LT_50_ values) of insecticides may be positively, negatively, or not influenced by temperature, and variation may be present between life stages and even during a bioassay [40]. The greatest effect of temperature for both insecticides and termite species was between 16 and 22 °C and for the lowest concentration (Table 1, Table 2, Table 3 and Table 4). For example, LT_50_ values for 0.5-ppm fipronil against the Formosan subterranean termite declined 70.5% between 16 and 22 °C, but did not change significantly (slope was not significantly different from 0) between 22 and 28 °C. For indoxacarb at 16 and 22 °C, there was a reduction in LT_50_ values of 42.7%. Over the entire temperature range and all concentrations there was no significant linear relationship between LT_50_ value and temperature. At higher concentrations, there was little variation in LT_50_ values, especially between 22 and 28 °C (Table 1, Table 2, Table 3 and Table 4).

Temperature may affect insecticide LT_50_ values by altering target site interactions, distribution, metabolism, and penetration [40]. Unfortunately, there is relatively little information on the toxicology of insecticides in termites. In general, enzymatic activity is greater at greater temperatures, which would result in faster detoxification with cytochrome P450, other oxidases, and esterases. Interestingly, the metabolism of fipronil results in the formation of a neurotoxic, oxidative sulfone metabolite that “retains essentially all the toxicity of its parent compound” [41]. Unlike fipronil, indoxacarb is considered a pro-insecticide that must be metabolized by esterases or amidases into its more active form [27]. Presumably, these enzymes are faster or more efficient at higher temperatures; however, LT_50_ values were not significantly affected by temperature particularly at 75 and 100 ppm (Table 1 and Table 3).

Intoxication, ataxia, and moribundity behaviors were observed for most concentration and temperature combinations of indoxacarb-exposed termites (Figure 3 and Figure 4) whereas ataxia was rarely observed in fipronil-exposed termites (Figure 1 and Figure 2). The sequence and percentage of behavioral symptoms may be related to the difference in homogeneity of response by termites to the insecticides (i.e., the slope of the log-time probit line). The mean slope for fipronil-exposed termites was 5.86 for eastern subterranean termites and 6.43 for Formosan subterranean termites (Table 1 and Table 2), whereas the slopes for indoxacarb-exposed termites were 2.80 and 2.26 for eastern and Formosan subterranean termites, respectively (Table 3 and Table 4). A more homogeneous response to insecticide-exposure (higher slopes) indicates relatively more kill as opposed to behavioral symptoms. This experimental design may have reduced our ability to detect behavioral symptoms by first, initiating observations at 4 h, and second by determining and averaging the behaviors of groups rather than following individual insects. It is likely that all behavioral symptoms would have been observed in fipronil-exposed termites at lower concentrations.

Insecticide-induced behaviors may be relevant to termite control. Formosan subterranean termites avoid dead nestmates killed with insecticides and rapidly wall off cadavers eliminating further exposure to the colony [42]. Dead termites may even be repellent to nestmates and reduce feeding [43]. Behavioral symptoms of insecticide exposure, especially intoxication (*sensu* Quarcoo et al. [34]), affect the period that insecticide-exposed termites are available to the colony. Rapid occurrence of behavioral symptoms such as ataxia and moribundity due to high concentrations and high temperatures could hinder worker termites’ ability to move far away from treatment [44], discontinue termiticide uptake [45], and act as a donor to transfer the termiticide actively to other colony members far away from the treatment [46]. However, sublethal behaviors such as ataxia and moribundity of exposed termites elicits grooming and caregiving from unexposed termites, enhancing the spread of insecticide within a colony through secondary and tertiary transfer [4,5].

Aguilar et al. [47] found that excavation of soil particles by ants was reduced by crowded conditions; however, when a portion of the group became idle, “traffic flow” and particle excavation increased. For termites, sublethal behaviors may similarly increase movement within tunnels facilitating contact with additional insecticide. Henderson [48] reported that delayed and/or long durations of toxicity symptoms increase the likelihood of transmission of insecticides to untreated nestmates by increasing the period of exposure. Similarly, Song and Hu [49] found that longer interaction times between donor and recipient termites resulted in greater mortality of recipients. Therefore, while fast acting insecticides provide immediate kill, the potential for spread within a colony is more limited. Slower acting or lower concentrations that kill, but induce prior behavioral symptoms may increase the potential for nestmate contact and ultimately kill off the entire colony.

## 5. Conclusions

This study demonstrated the effects of temperature and non-repellent insecticide concentration on the behavior and mortality of eastern, *Reticulitermes flavipes* (Kollar), and Formosan, *Coptotermes formosanus* Shiraki, subterranean termites. Behavioral symptoms associated with insecticide exposure including intoxication, ataxia, and moribundity [34] were observed for both species at most insecticide concentration and temperature combinations. The faster acting the temperature/concentration combination (lower LT_50_ value), the lower the number/variety of behavioral symptoms observed. For fipronil, LT_50_ values declined with increasing concentrations and temperatures for both eastern and Formosan subterranean termites. Contrarily, LT_50_ values for indoxacarb were not always highest at the lowest temperature (16 °C), and the indoxacarb effects were similar for both termite species. While higher temperatures and insecticide concentrations kill individual termites more rapidly, movement and behavioral symptoms of insecticide-exposed termites may actually facilitate transfer of insecticides throughout a colony.

## Figures and Tables

**Table 1:** Effect of concentration and temperature on mortality of eastern subterranean termites, *R. flavipes*, exposed to fipronil.

Concentration (ppm)	Temperature (°C)	LT_50_ (h)	95% FL (h)	χ^2^	Slope ± SE
0.5	16	84.92	71.47–99.88	91.893	1.77 ± 9.18
0.5	22	26.84	24.66–28.95	138.107	5.79 ± 0.49
0.5	28	20.14	18.65–21.58	181.186	5.45 ± 0.40
1.0	16	23.65	22.73–24.55	257.079	8.90 ± 0.55
1.0	22	16.68	15.78–17.54	105.292	8.89 ± 0.87
1.0	28	11.36	9.89–12.81	108.114	4.17 ± 0.40
5.0	16	22.97	17.80–27.51	96.070	2.85 ± 0.29
5.0	22	15.14	14.57–15.68	119.117	13.36 ± 1.22
5.0	28	0.96	0.37–1.72	56.049	1.60 ± 0.21

**Table 2:** Effect of concentration and temperature on mortality of Formosan subterranean termites, *C. formosanus*, exposed to fipronil.

Concentration (ppm)	Temperature (°C)	LT_50_ (h)	95% FL (h)	χ^2^	Slope ± SE
0.5	16	98.78	83.65–115.78	69.564	2.46 ± 0.29
0.5	22	29.12	27.33–30.83	135.573	7.54 ± 0.65
0.5	28	19.20	16.34–21.90	87.952	3.96 ± 0.42
1.0	16	31.73	26.31–37.10	194.653	1.96 ± 0.14
1.0	22	23.16	22.92–23.39	3.61 × 10^5^	114.44 ± 0.00
1.0	28	8.57	7.28–9.84	94.090	3.79 ± 0.39
5.0	16	22.48	17.80–26.97	78.932	3.30 ± 0.37
5.0	22	21.91	21.55–22.01	0.000	212.93 ± 0.00
5.0	28	2.02	1.06–2.98	51.732	2.14 ± 0.30

**Table 3:** Effect of concentration and temperature on mortality of eastern subterranean termites, *R. flavipes*, exposed to indoxacarb.

Concentration (ppm)	Temperature (°C)	LT_50_ (h)	95% FL (h)	χ^2^	Slope ± SE
50.0	16	43.52	42.30–44.75	292.324	9.60 ± 0.56
50.0	22	17.33	12.56–22.26	90.377	1.99 ± 0.21
50.0	28	6.42	4.47–8.41	114.016	1.87 ± 0.18
75.0	16	23.31	16.44–30.50	79.284	1.80 ± 0.20
75.0	22	16.65	11.45–22.03	90.470	1.67 ± 0.18
75.0	28	16.91	12.26–21.72	92.779	1.94 ± 0.20
100.0	16	20.52	14.44–27.01	65.450	2.19 ± 0.28
100.0	22	16.91	12.29–21.71	86.538	2.10 ± 0.23
100.0	28	7.57	5.88–9.30	155.352	2.03 ± 0.16

**Table 4:** Effect of concentration and temperature on mortality of Formosan subterranean termites, *C. formosanus*, exposed to indoxacarb.

Concentration (ppm)	Temperature (°C)	LT_50_ (h)	95% FL (h)	χ^2^	Slope ± SE
50.0	16	69.1	63.94–74.16	286.697	4.32 ± 0.26
50.0	22	39.62	26.40–54.52	44.193	1.85 ± 0.28
50.0	28	37.69	23.22–54.89	31.559	2.10 ± 0.37
75.0	16	29.94	20.21–40.62	51.207	2.03 ± 0.28
75.0	22	28.33	19.28–38.21	52.846	2.06 ± 0.28
75.0	28	27.14	18.49–36.55	54.090	2.04 ± 0.28
100.0	16	28.34	19.52–37.83	60.040	1.93 ± 0.25
100.0	22	31.25	21.04–42.45	50.172	1.97 ± 0.28
100.0	28	25.28	17.90–33.38	67.110	2.08 ± 0.25
